# Uropathogenic *Escherichia coli* wield enterobactin-derived catabolites as siderophores

**DOI:** 10.1016/j.jbc.2023.105554

**Published:** 2023-12-10

**Authors:** Zongsen Zou, John I. Robinson, Lindsey K. Steinberg, Jeffrey P. Henderson

**Affiliations:** 1 Center for Women’s Infectious Diseases Research, Washington University School of Medicine, St Louis, Missouri, USA; 2 Division of Infectious Diseases, Department of Internal Medicine, Washington University School of Medicine, St Louis, Missouri, USA

**Keywords:** *Escherichia coli*, iron, siderophore, metabolomics, exometabolome, enterobactin, Gram-negative bacteria

## Abstract

Uropathogenic *Escherichia coli* (UPEC) secrete multiple siderophore types to scavenge extracellular iron(III) ions during clinical urinary tract infections, despite the metabolic costs of biosynthesis. Here, we find the siderophore enterobactin (Ent) and its related products to be prominent components of the iron-responsive extracellular metabolome of a model UPEC strain. Using defined Ent biosynthesis and import mutants, we identify lower molecular weight dimeric exometabolites as products of incomplete siderophore catabolism, rather than prematurely released biosynthetic intermediates. In *E. coli*, iron acquisition from iron(III)–Ent complexes requires intracellular esterases that hydrolyze the siderophore. Although UPEC are equipped to consume the products of completely hydrolyzed Ent, we find that Ent and its derivatives may be incompletely hydrolyzed to yield products with retained siderophore activity. These results are consistent with catabolic inefficiency as means to obtain more than one iron ion per siderophore molecule. This is compatible with an evolved UPEC strategy to maximize the nutritional returns from metabolic investments in siderophore biosynthesis.

Urinary tract infections (UTIs) are among the most common outpatient and inpatient infections encountered by physicians (1, 2, 3, 4). *Escherichia coli* is the bacterial species most commonly associated with UTI, accounting for about 70 to 95% of clinical cases (5, 6). Clinical *E. coli* isolates associated with UTI that exhibit polymorphisms in conserved genes (7, 8, 9) and carry accessory genes associated with increased pathogenic potential are designated as uropathogenic *E. coli* (UPEC) (2, 4, 10). Prominent among these virulence-associated adaptions are iron uptake systems, such as siderophores, which use distinctive chemical groups to competitively bind iron and render it selectively bioavailable to support bacterial growth (2, 3, 10, 11, 12, 13, 14). In UPEC, siderophore iron-acquisition systems have been identified as both colonization and virulence factors during UTI pathogenesis (15, 16, 17, 18, 19). The enterobactin (Ent), salmochelin, yersiniabactin, and aerobactin siderophore systems have all been associated with *E. coli* strains causing extraintestinal infections (20, 21, 22).

Siderophores are specialized secreted metabolites (exometabolites) that are synthesized by nonessential bacterial pathways and competitively chelate extracellular iron(III) during the iron-limited growth conditions characteristic of infection microenvironments (16, 17, 23, 24). The resulting iron(III)–siderophore complexes are selectively imported by bacterial transporters as an iron source. *E. coli* and many other Gram-negative bacteria actively transport iron–siderophore complexes through outer membrane receptors using the cytoplasmic membrane–localized TonB–ExbB–ExbD complex, which transduces energy from the proton motive force (25, 26, 27). Siderophore biosynthesis and transport systems are regulated by the ferric uptake regulator, a transcriptional repressor that downregulates siderophore gene transcription in conditions associated with high cytosolic iron (28).

All UPEC carry the conserved Ent system and may encode up to three additional siderophore systems, each associated with chemically distinctive exometabolomes (29, 30, 31). Biosynthesis of these additional exometabolites incurs additional metabolic demands (32), suggesting that their sustained presence in clinical populations is associated with siderophore-specific payoffs. For example, the salmochelin system, encoded by genes in the *iroA* cassette, glucosylates Ent to improve its aqueous solubility and evade sequestration by the host immune protein lipocalin-2–siderocalin–NGAL (14, 33, 34). The yersiniabactin system in UPEC supports multiple nonsiderophore functions not associated with Ent or salmochelin (35, 36). Yersiniabactin production incurs metabolic costs, which appear to be mitigated by an ability to recycle the intact siderophore to support multiple rounds of metal ion import (37) and an additional quorum-sensing regulatory input that emphasizes biosynthesis in diffusionally restricted or crowded environments where the siderophore is more likely to remain nearby (38).

Ent is detectable in the urine of patients with UTIs, where its synthesis is required to evade growth inhibition by lipocalin-2 (13, 14). Ent achieves exceptional iron(III) affinity (*K*_*d*_ ≈ 10^−52^ M) with three catechol (1,2-dihydroxybenzene) groups that provide all six coordination sites for iron(III) (10, 39). Ent is synthesized by a nonribosomal peptide synthetase system encoded by *entABCDEF*. This nonribosomal peptide synthetase system is a molecular assembly line that synthetizes Ent by repeatedly forming enzyme-bound *N*-(2,3-dihydroxybenzoyl)serine (DHBS) and linking them *via* ester bonds until a cyclic trilactone core composed of three DHBS is released (40, 41). In UPEC expressing the *iroA* cassette, the glucosyltransferase IroB further modifies Ent catechols with up to three distinctive C-linked glucoses (10, 42). Iron retrieval from imported iron(III) Ent complexes (with or without C-glucose modifications) requires dissociation through both esterase-catalyzed Ent hydrolysis (by Fes and/or IroD) and iron(III) reduction to iron(II) (43, 44, 45).

In this study, we examined the Ent biosynthetic pathway’s contribution to the iron-dependent UPEC exometabolome. We measured targeted mutant strains and chemical complementation with purified products to assess the catabolic origins of short-length catechol exometabolites. To assess the nutritional potential of siderophore catabolism, we used reverse stable isotope labeling to find that 2,3-dihydroxybenzoic acid (DHB) from outside the biosynthetic pathway could be used for Ent biosynthesis. Finally, we used a siderophore-dependent growth condition to evaluate the siderophore potential of nontrimeric Ent metabolites found in the UPEC exometabolome. Our findings are consistent with a catabolic network that has evolved to maximize the iron delivery potential of Ent biosynthesis.

## Results

### Ent and the iron-responsive exometabolome in UPEC

**Figure 1:**
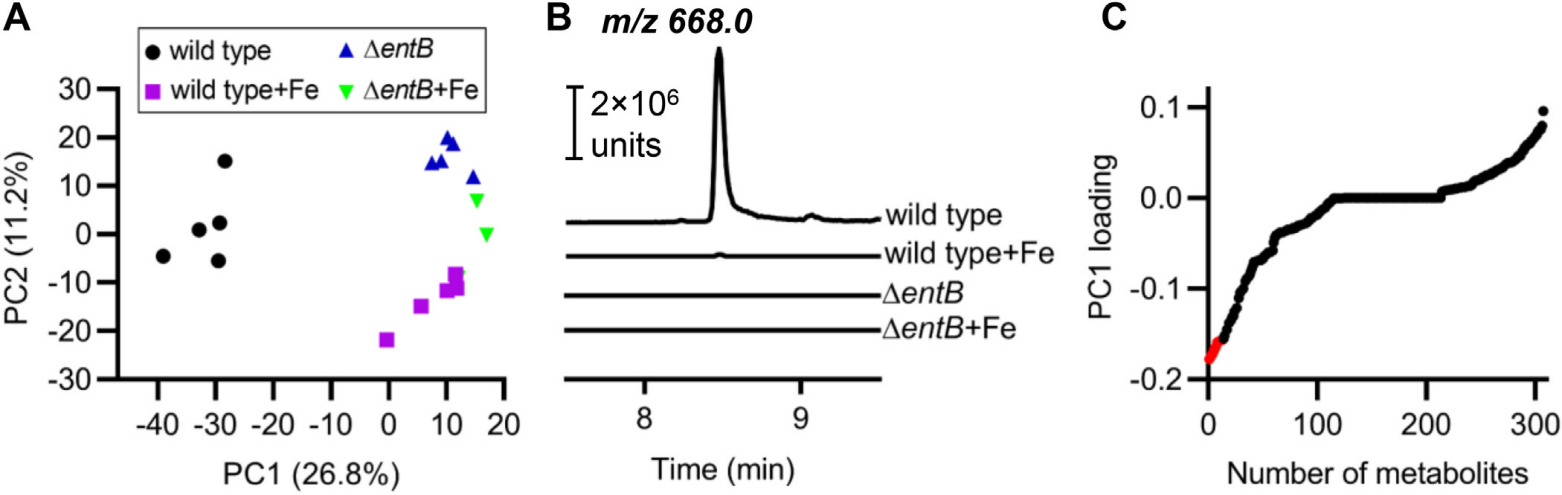
**The enterobactin (Ent) biosynthetic pathway is a prominent contributor to the iron-responsive uropathogenic *Escherichia coli* UTI89 exometabolome.** Sparse principal component analysis (sPCA) was performed to identify LC–MS exometabolome profiles that distinguish four groups of conditioned media: UTI89 grown in low and high iron (addition of 100 μM FeCl_3_) media (wildtype and wildtype + Fe, respectively) and the Ent-null mutant UTI89Δ*entB* grown in low and high iron media (*entB* and *entB* + Fe, respectively). *A*, the score plot depicts each replicate LC–MS exometabolome (as a data point) as a function of principal components 1 and 2 (PC1, PC2), which are the first and second most influential modes of exometabolomic variation across all specimens. The combination of exometabolites comprising the PC1 axis separate the wildtype, low iron condition from the other conditions. *B*, LC–MS/MS chromatograms corresponding to the precursor–product ions from Ent (*m/z* 668.0) for each experimental group. Chromatograms are displayed in identical ion current unit scales. *C*, a PC1 loading plot displays the contribution of each LC–MS exometabolite feature to the PC1 value. The 13 most influential metabolites with greater abundance in wildtype UTI89 (lower PC1 value) are identified as *red data points*.

To define the iron-responsive exometabolome of UPEC and its relationship to the *ent-*encoded biosynthetic pathway, we compared small molecule profiles in conditioned media from the model UPEC strain UTI89 and its isogenic biosynthesis-deficient mutant, UTI89Δ*entB* (21), in low and high iron conditions (32) using LC–MS. Sparse principal component analysis (sPCA) was performed on these data to determine whether exometabolite composition distinguishes the different conditioned media. sPCA yields a series of principal components (PCs), mathematical terms that are a series of independent linear combinations of features associated with feature variability between specimens. Here, PC1 is a mathematical expression of exometabolites comprising the largest mode of exometabolomic variation (26.8% of total variation) between conditioned media, with PC2 being the next largest, and so forth (Figs. 1*A* and S1*A*). In a two-dimensional score plot of PC2 *versus* PC1, exometabolomes of low iron media conditioned by wildtype UTI89 formed a discrete cluster of values separated along PC1 from the profiles of other conditions. This is the greatest group-wise separation among the conditioned examined and is consistent with a distinctive iron-responsive exometabolome in UTI89 dominated by *ent*-associated biosynthetic products. Logistic regression of PC1 values to classify these two PC1 exometabolome clusters yielded a prediction accuracy of 1.0 (SD = 0, Fig. S1*B*) and an area under the receiver operating characteristic curve of 1.0 (SD = 0, Fig. S1*C*) with fourfold crossvalidation. PC1 differences did not correspond to intergroup differences in growth density (Fig. S2). The distinctive PCA grouping of wildtype UTI89 grown in low iron corresponds with detection of Ent, the canonical eponymous product of the Ent biosynthesis pathway (Fig. 1*B*). Together, these results are consistent with a prominent role for the Ent biosynthetic pathway in defining the iron-responsive UTI89 exometabolome.

### Multiple Ent-associated products define the UTI89 exometabolome

**Figure 2:**
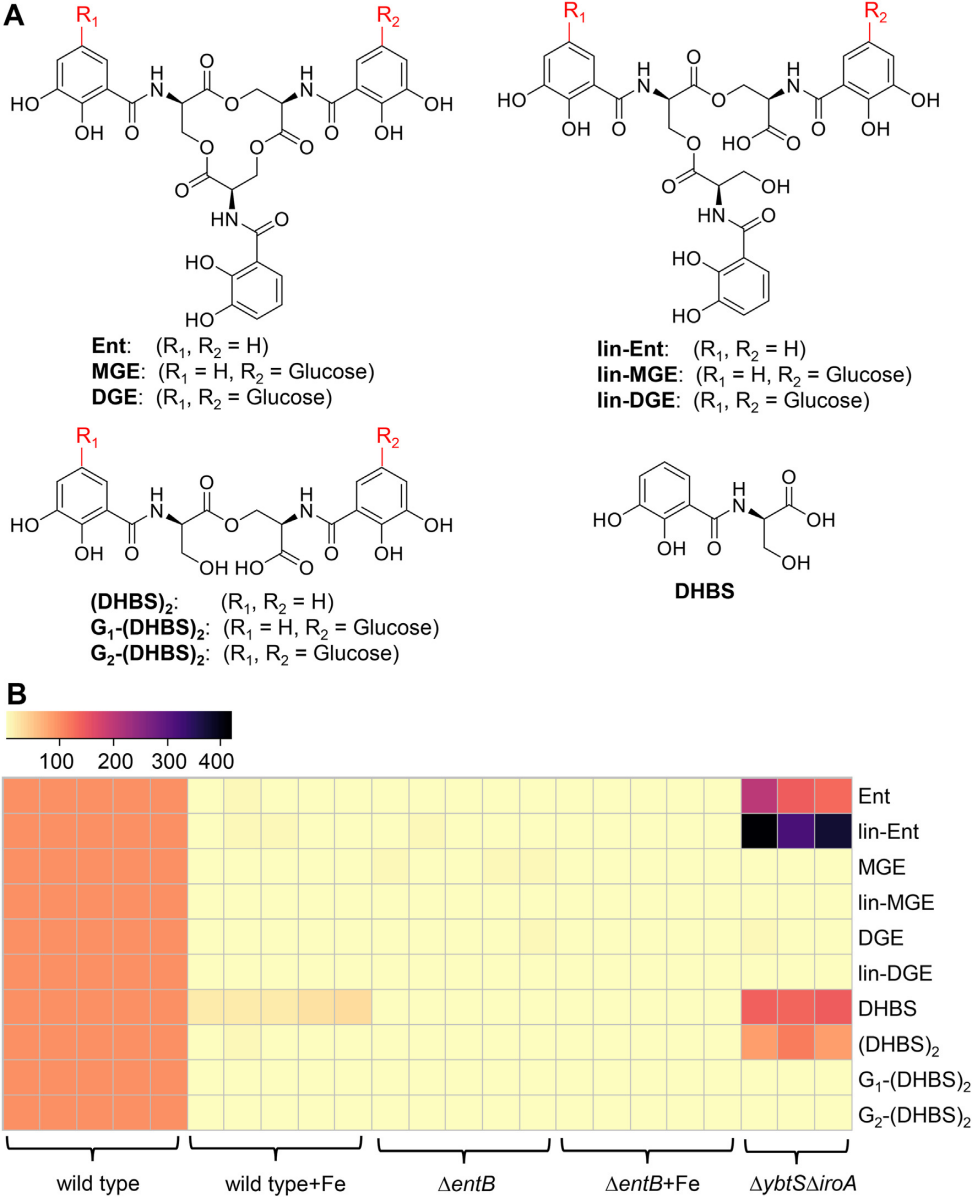
**Exometabolites associated with the iron-responsive UTI89 exometabolome**. *A*, chemical structures of the 10 enterobactin (Ent)-associated exometabolites identified by comparative metabolomic analysis, including Ent, monoglucosylated Ent (MGE), diglucosylated Ent (DGE), linear Ent (lin-Ent), linear monoglucosylated Ent (lin-MGE), linear diglucosylated Ent (lin-DGE), *N*-(2,3-dihydroxybenzoyl)serine dimer [(DHBS)_2_], monoglucosylated *N*-(2,3-dihydroxybenzoyl)serine dimer [G_1_-(DHBS)_2_], diglucosylated *N*-(2,3-dihydroxybenzoyl)serine dimer [G_2_-(DHBS)_2_], and *N*-(2,3-dihydroxybenzoyl)serine monomer (DHBS). The positions of C-glucosylated DHBS units within linear polymers have not been definitively identified. *B*, heatmap showing Ent-associated exometabolite concentrations in media with iron supplementation or defined biosynthetic mutants of UTI89. Intensity represents concentration expressed as ratio of LC–MS/MS peak area to that of internal standard. Individual biological replicates are shown for each condition.

The *ent-*associated exometabolites that define PC1 are of interest and may be identified by loading analysis, which identifies the magnitude of each exometabolite’s contribution (the loading) to a PC value. For PC1, loading analysis identifies multiple exometabolites contributing to PC1 (Fig. 1*C*). Detailed mass spectrometric and chromatographic analyses of the 13 molecular features with the largest PC1 loadings associated with the UTI89 exometabolome under iron-restricted conditions (Figs. S3–S12) identified a series of 10 DHBS polymers (Fig. 2*A*) consistent with Ent and salmochelin biosynthesis (46, 47, 48) (Table S1). These included cyclic and linear DHBS trimers with 0, 1, or 2 C-glucosylations, DHBS dimers with 0, 1, or 2 C-glucosylations, and monomeric DHBS previously reported in an avian pathogenic *E. coli* strain. Unlike the avian pathogenic *E. coli* strain, UTI89 did not produce triglucosylated Ent products (49), consistent with interstrain differences in the Ent exometabolome that are not explained by *iroA* alone. To more precisely quantify these exometabolites, we constructed a high-resolution targeted LC–MS/MS multiplexed selected reaction monitoring (LC–MRM) method (Table 1). We confirmed that all 10 products were present in low iron media conditioned by wildtype UTI89, were significantly diminished in high iron media conditioned by UTI89, and were undetectable in any media conditioned by UTI89Δ*entB* (Figs. 2*B*, S13, and S14, *p* < 0.001). In an *iroA-*null strain (UTI89Δ*ybtS*Δ*iroA*) that lacks the C-glucosylation pathway, C-glucosylated exometabolites were absent, whereas nonglucosylated exometabolites were elevated, (Figs. 2*B* and S14, *p* < 0.001), consistent with the precursor–product relationship between these exometabolites. Together, these results connect iron-associated biosynthetic activity in UPEC to multiple Ent-related exometabolites extending beyond the canonical trimeric DHBS products.

**Table 1:** Targeted LC–MS/MS protocols for detecting and quantifying 10 Ent siderophores and short-length products

Identity	Selected ions (m/z)		Retention time (min)	References
	Q1^a^	Q3^b^		
Ent	668	222	8.47	(21, 46, 51, 86, 90, 91)
lin-Ent	686	222	7.21	(21, 46, 47, 51)
MGE	832	222	6.94	(46, 47, 51)
lin-MGE	848	222	5.89	(46, 47, 51)
DGE	993	222	5.95	(46, 47, 51, 92)
lin-DGE	1010	222	4.98	(21, 46, 47, 51, 92)
(DHBS)_2_	464	222	5.92	(46, 51, 86, 90, 91)
G_1_-(DHBS)_2_	625	222	4.69	(46, 47, 51, 92)
G_2_-(DHBS)_2_	787	402	3.64	(46, 47, 51)
DHBS	240	153	2.34	(46, 51, 86, 90, 91)

a Q1 represents the *m/z* of the precursor ion selected by the first quadrupole during MS/MS.

b Q3 represents the *m/z* of the fragment ion selected by second quadrupole during MS/MS.

### Outer membrane importers differentially affect Ent-associated exometabolites

**Figure 3:**
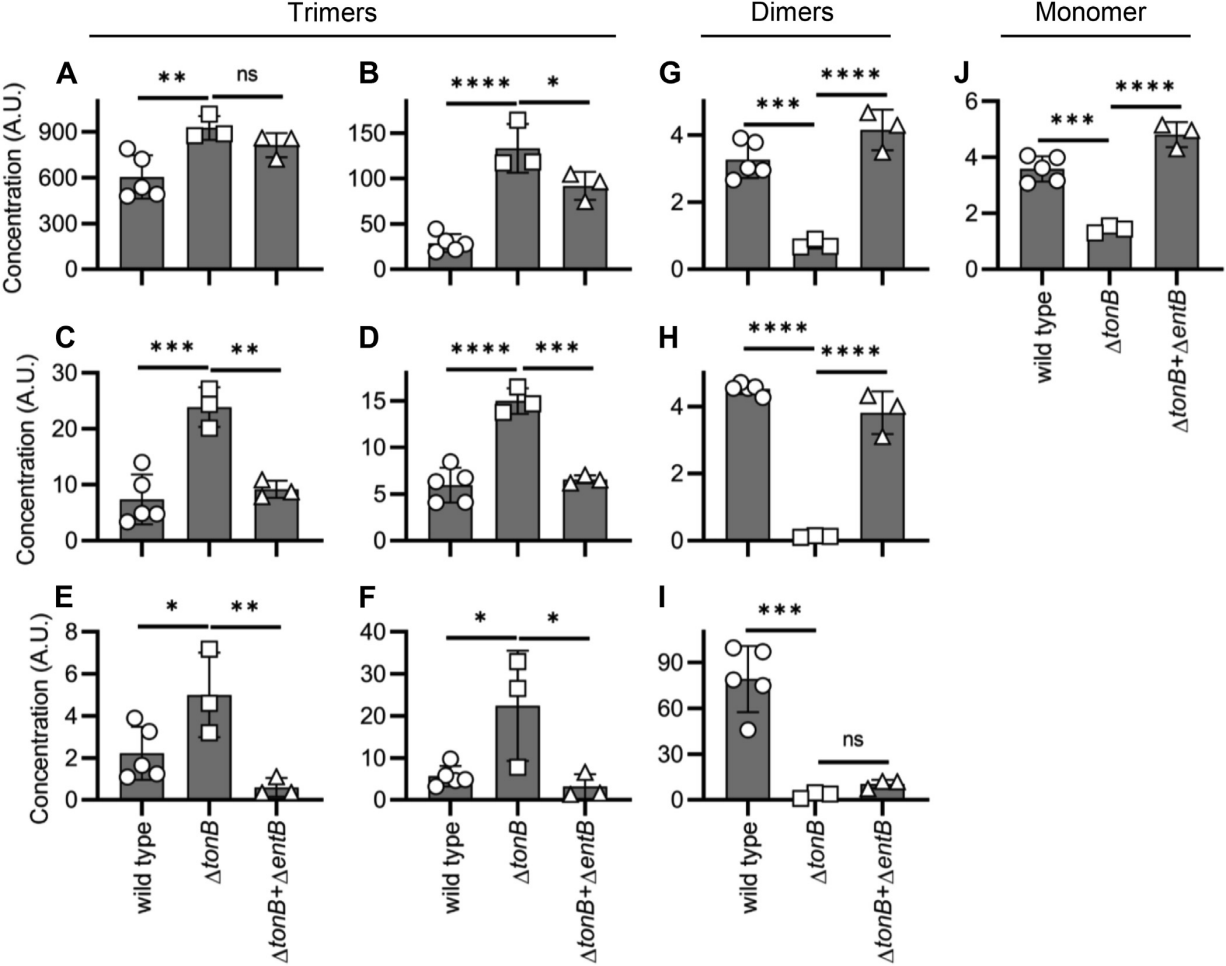
**Outer membrane import differentially affects trimeric and nontrimeric enterobactin (Ent)-associated exometabolites in culture.** Ent-associated exometabolite concentrations in media conditioned by UTI89 (wildtype), an import-deficient UTI89 mutant (Δ*tonB*), or coculture of Ent-null and import-deficient UTI89 mutants (Δ*tonB* + Δ*entB*). *Y-*axis is concentration expressed as ratio of LC–MS/MS peak area to that of internal standard. (*A*) Ent. (*B*) lin-Ent. (*C*) MGE. (*D*) lin-MGE. (*E*) DGE. (*F*) lin-DGE. (*G*) (DHBS)_2_. (*H*) G_1_-(DHBS)_2_. (*I*) G_2_-(DHBS)_2_. (*J*) DHBS. Data are presented as mean ± SD with at least three biologically independent samples. Statistics were performed using one-way ANOVA with Dunnett’s multiple-comparison test with *p* ≤ 0.05 considered as statistically significant. ns, not significant; ∗*p* ≤ 0.05, ∗∗*p* < 0.01, ∗∗∗*p* < 0.001, ∗∗∗∗*p* < 0.0001.

While trimer products are consistent with the Ent biosynthetic pathway, the specific origin of short-length dimeric and monomeric products, (DHBS)_2_, G_2_-(DHBS)_2_, G_1_-(DHBS)_2_, and DHBS is unclear. We considered that these truncated products could reflect premature release from the biosynthetic pathway (anabolic production) (11, 50), spontaneous extracellular hydrolysis, or intracellular esterolysis of imported ferric catechol siderophores (catabolic production) (46, 47, 51). To distinguish these possibilities, we compared UTI89 with UTI89Δ*tonB*, an isogenic mutant with a deficiency in siderophore import at the outer membrane. In *E. coli* and related Gram-negative bacteria, the TonB–ExbB–ExbD complex energizes outer membrane transporters to import ferric siderophores (52). Relative to UTI89, UTI89Δ*tonB* cultures exhibit a strikingly dichotomous effect on Ent-associated exometabolites, with elevated trimer concentrations and diminished dimer and monomer concentrations (Fig. 3). These differences were not associated with differential growth density between groups (Fig. S15). These results are consistent with intracellular dimer and monomer production in events that are downstream from extracellular trimer import.

### Coculture with import-proficient UTI89 complements the UTI89ΔtonB phenotype

To further test the hypothesis that monomer and dimer exometabolites are products of siderophore catabolism, we devised a coculture system in which UTI89Δ*tonB* is poised to serve as a siderophore producer and Ent-deficient UTI89Δ*entB* as a siderophore consumer.

We hypothesized that UTI89Δ*entB* import of UTI89Δ*tonB*-derived exometabolites would counteract the UTI89Δ*tonB* dimer and monomer deficiency phenotype. Compared with UTI89Δ*tonB*-conditioned media, media conditioned by the UTI89Δ*tonB* + UTI89Δ*entB* coculture contained significantly greater monomer and dimer concentrations and variably lower trimer concentrations (Fig. 3). As such, the combined *ent* exometabolome of UTI89Δ*tonB* + UTI89Δ*entB* more closely resembled that of wildtype UTI89 than either mutant alone. Different levels of Ent-associated products were not associated with growth density differences between groups (Fig. S15). These results are consistent with extracellular UTI89Δ*tonB*-derived trimers as public goods that are imported by UTI89Δ*entB*, which partially catabolizes them and releases esterolysis products to the extracellular space (47, 51).

### Monomer and dimer production during trimer-dependent growth

**Figure 4:**
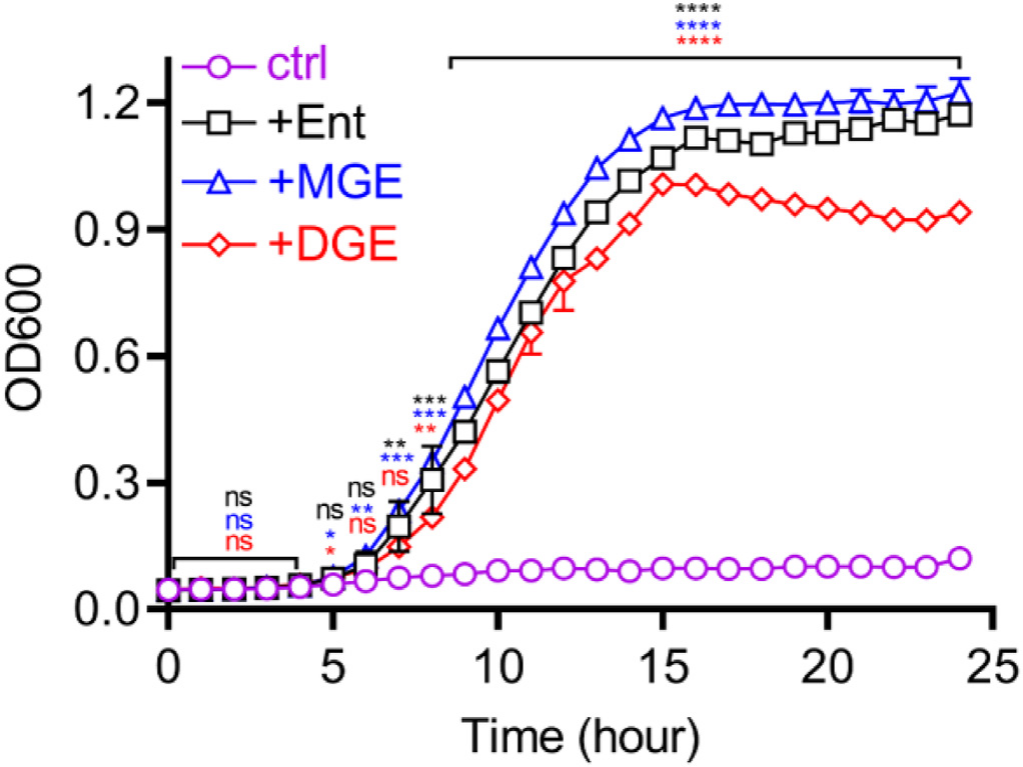
**Trimer supplementation supports siderophore-null UTI89 mutant growth in siderophore-dependent growth medium.** Growth of the siderophore-null strain UTI89Δ*entB*Δ*ybtS* was measured by absorbance at 600 nm in siderophore-dependent medium following supplementation with Ent, MGE, or DGE and compared with unsupplemented control (ctrl). Statistics were performed using one-way ANOVA with Dunnett’s multiple-comparison test with *p* ≤ 0.05 considered as statistically significant. ns, not significant; ∗*p* ≤ 0.05, ∗∗*p* < 0.01, ∗∗∗*p* < 0.001, and ∗∗∗∗*p* < 0.0001.

**Figure 5:**
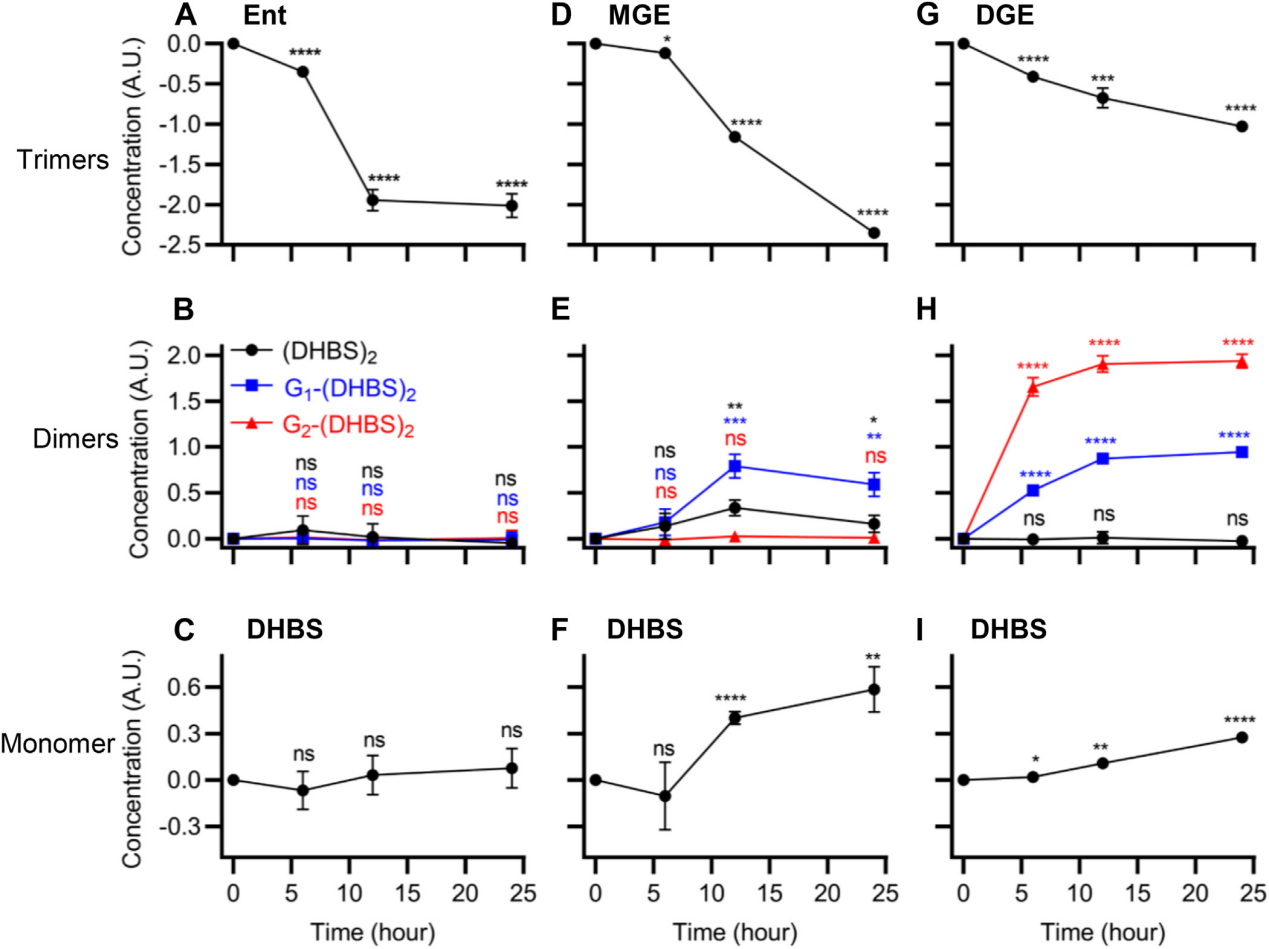
**Enterobactin (Ent)-associated exometabolites during trimer-dependent growth.** Siderophore-null strain UTI89Δ*entB*Δ*ybtS* was cultured in siderophore-dependent medium containing purified Ent, MGE, or DGE. The Ent-associated metabolome in the medium was measured using LC–MS/MS at time points during culture. *A*–*C*, Ent (*A*) is imported and catabolized by UTI89Δ*entB*Δ*ybtS* without producing any dimer (*B*) or monomer (*C*) *ent* catechol compounds. *D*–*F*, MGE (*D*) is imported and catabolized by UTI89Δ*entB*Δ*ybtS*, which produces (DHBS)_2_ and G_1_-(DHBS)_2_ dimers (*E*) and DHBS monomer (*F*). *G*–*I*, DGE (*G*) is imported and catabolized by UTI89Δ*entB*Δ*ybtS*, which produces G_1_-(DHBS)_2_ and G_2_-(DHBS)_2_ dimers (*H*) and DHBS monomer (*I*). Statistics were performed using unpaired *t* test with *p* ≤ 0.05 considered as statistically significant. ns, not significant; ∗*p* ≤ 0.05, ∗∗*p* < 0.01, ∗∗∗*p* < 0.001, and ∗∗∗∗*p* < 0.0001.

Ent-associated trimers contain two or three serine–serine ester bonds and three serine–DHB peptide bonds (Fig. 2*A*) with potential for hydrolysis to yield free DHB and serine, which may become new metabolic substrates in the cytoplasm. Despite this catabolic potential, UTI89 releases incompletely hydrolyzed trimer catabolites. To determine whether this occurs during siderophore-dependent growth, we measured the Ent-associated exometabolomes of siderophore-null UTI89 (UTI89Δ*entB*Δ*ybtS*) cultures with trimer supplementation. Growth of this strain was rendered siderophore dependent by addition of bovine serum albumin, a biologically relevant nonspecific binder of labile iron ions (53, 54). Compared with siderophore-free controls, Ent, MGE, or DGE addition stimulated UTI89Δ*entB*Δ*ybtS* growth (Fig. 4) and were progressively consumed during culture (Fig. 5, *A*, *D*, and *G*), consistent with their canonical siderophore activity. Dimer and monomer production varied with the specific trimer provided. Ent supplementation yielded neither dimer nor monomer (Fig. 5, *B* and *C*), MGE supplementation yielded (DHBS)_2_, G_1_-(DHBS)_2_, and DHBS (Fig. 5, *E* and *F*), and DGE yielded G_1_-(DHBS)_2_, G_2_-(DHBS)_2_, and DHBS (Fig. 5, *H* and *I*). Dimer C-glucosylation products are structurally consistent with the C-glucosylation structure of each trimeric substrate. These results are consistent with dimer and monomer production from esterolysis following cyclic trimer-mediated iron delivery. The lack of dimer or monomer generation from Ent is unexpected based on production by Ent-producing UTI89Δ*ybtS*Δ*iroA* (Fig. 2*B*). The nature of this discrepancy is unclear and may arise from unappreciated catabolic differences, regulatory pathways, or intracellular trafficking connected to these different strains, the different culture conditions, or combinations thereof.

### UTI89 uses exogenous DHB to synthesize Ent

**Figure 6:**
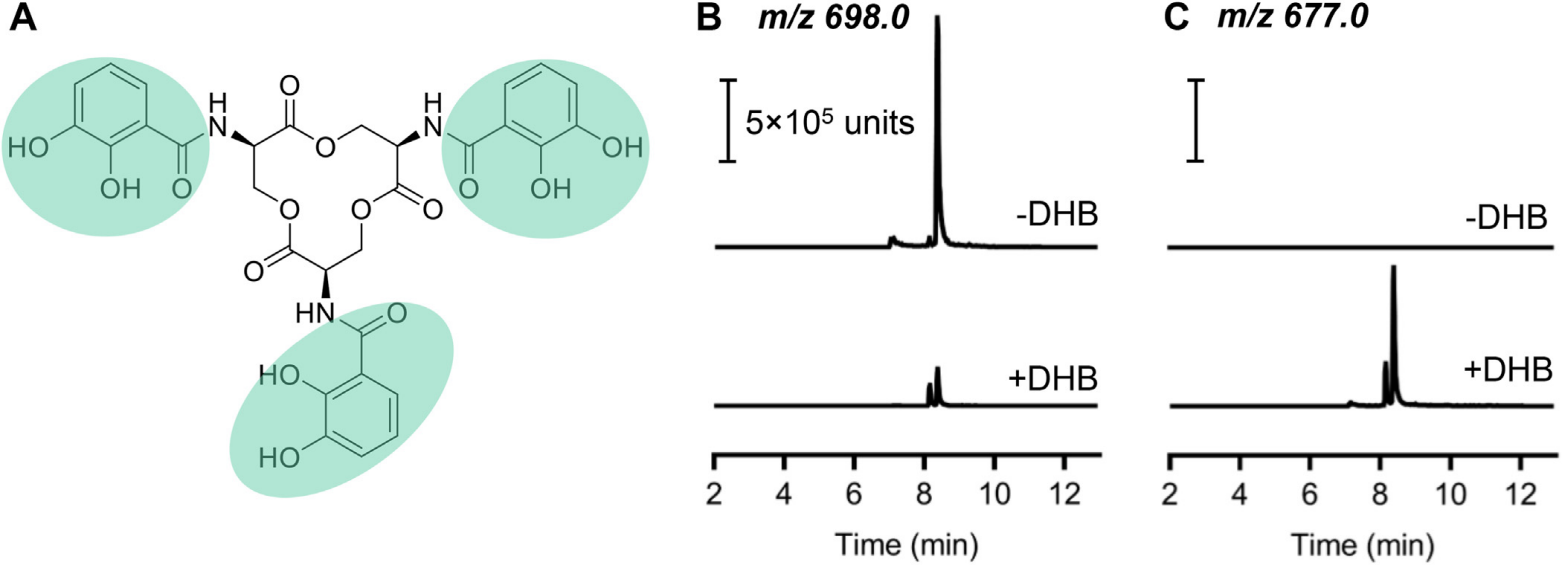
**Exogenous 2,3-dihydrobenzoic acid (DHB) supports enterobactin (Ent) biosynthesis.***A*, chemical structure of cyclic Ent with the three DHB-derived groups, containing seven carbon atoms, highlighted in *green*. *B*, LC–MS/MS detection of fully ^13^C_30_-substituted Ent ([M-H]^−^, *m/z* 698) in UTI89-conditioned ^13^C_3_-glycerol culture medium without (−DHB) or with (+DHB), 200 μM unlabeled DHB. *C*, LC–MS/MS detection of ^13^C_9_-substituted Ent ([M-H]^−^, *m/z* 677) into which three DHB molecules have been incorporated without (−DHB) or with (+DHB), 200 μM unlabeled DHB.

It is unclear why UTI89 foregoes complete catabolic reclamation of intracellular trimer constituents to instead release incompletely hydrolyzed trimer catabolites to the extracellular space. Bonacorsi *et al.* (55) have connected enhanced bacterial DHB production for siderophore biosynthesis as a virulence-associated activity in neonatal meningitis–associated *E. coli*, suggesting that UPEC could similarly benefit from DHB reclamation. To determine whether UPEC can use exogenously derived DHB to support trimer biosynthesis, we derived an experimental system to monitor its incorporation. Specifically, we used a reverse isotope-labeling strategy to detect incorporation of unlabeled carbon atoms from exogenous DHB during culture with ^13^C_3_-glycerol as the carbon source. We found that addition of 200 μM DHB led to the appearance of a new Ent isotopolog with an *m/z* value 21 atomic mass units lower than ^13^C-substituted Ent, consistent with ^12^C_7_-DHB incorporation at all three catechol sites (Fig. 6 and Table S2). DHB supplementation also yielded lower levels of singly and doubly substituted isotopologs that are 7 and 14 atomic mass units lower, respectively (Fig. S16 and Table S2). These results expand upon previous findings that the Ent deficiency of *entA*-deficient K12 *E. coli* cultures could be reversed by media supplementation with DHB (56, 57). Direct incorporation of an isotopically distinctive precursor shows that a UPEC strain Ent biosynthetic pathway can directly incorporate DHB from a nonendogenous source. This suggests that DHB incorporation is not limited to a tightly compartmented intracellular site supplied exclusively by endogenous biosynthesis. Analogous incorporation of exogenously supplied ^13^C-labeled 2-hydroxybenzoic acid or 2-aminobenzoic acid into the exometabolites yersiniabactin and escherichelin has been previously observed (11, 58), suggesting a generalized aromatic metabolite scavenging potential in UPEC. These observations are consistent with the potential metabolic value of complete Ent hydrolysis in UPEC.

### Siderophore activity of purified dimers

**Figure 7:**
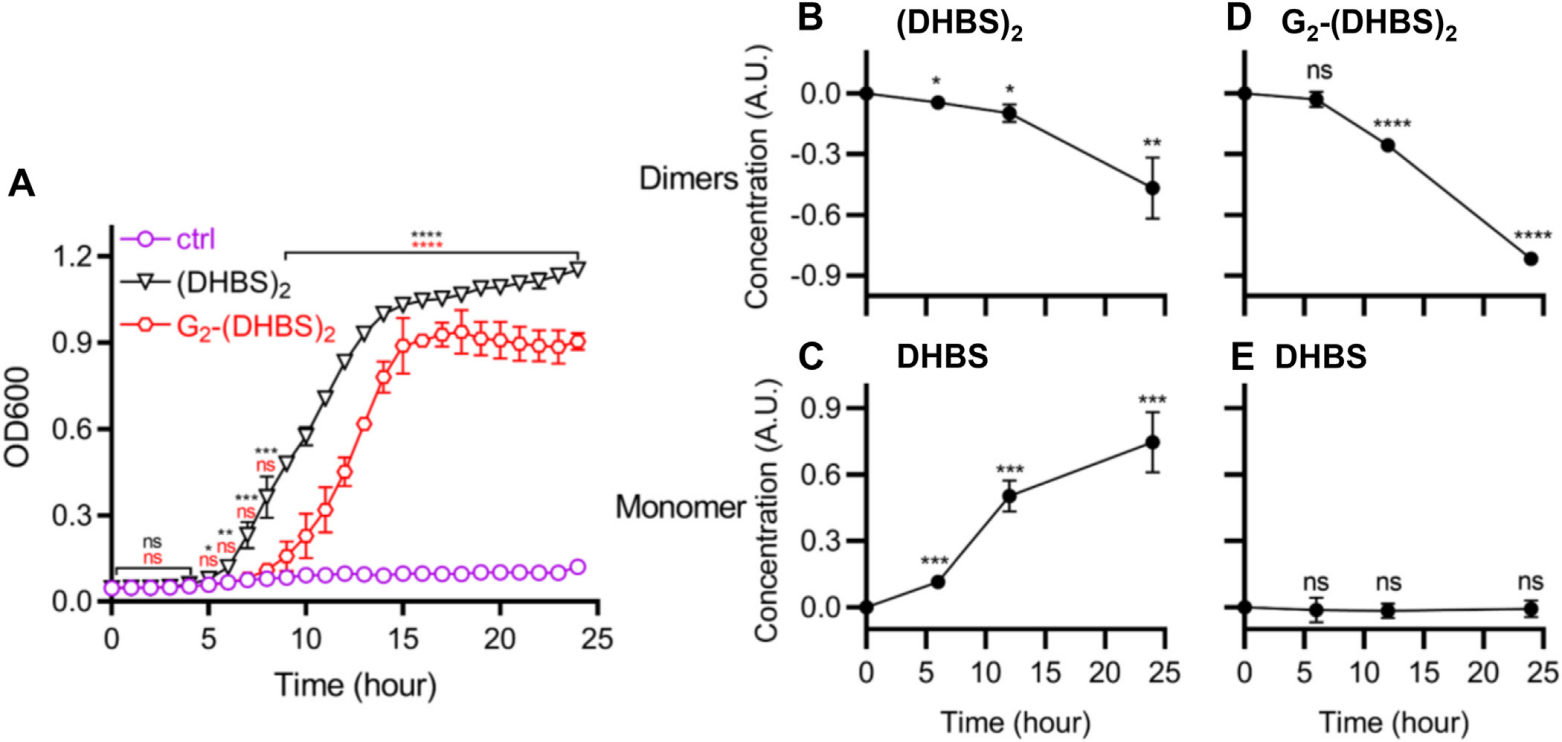
**Enterobactin (Ent)-associated dimers support siderophore-dependent growth**. *A*, growth of the siderophore-null strain UTI89Δ*entB*Δ*ybtS* was measured by absorbance at 600 nm in siderophore-dependent medium following supplementation with the Ent-associated dimer exometabolites (DHBS)_2_ or G_2_-(DHBS)_2_ or siderophore-free control (ctrl). *B*–*E*, the Ent-associated metabolome in the medium was measured using LC–MS/MS at time points during culture with dimer and monomer results shown for (DHBS)_2_-supplemented cultures (*B* and *C*) and G_2_-(DHBS)_2_-supplemented cultures (*D* and *E*). Statistics were performed using unpaired *t* test with *p* ≤ 0.05 considered as statistically significant. ns, not significant; ∗*p* ≤ 0.05, ∗∗*p* < 0.01, ∗∗∗*p* < 0.001, and ∗∗∗∗*p* < 0.0001. DHBS, DHBS, *N*-(2,3-dihydroxybenzoyl)serine.

We hypothesized that UPEC forego complete trimer hydrolysis because the resulting dimers retain valuable siderophore activity. This would enable biosynthesis of one trimer molecule to support multiple rounds of iron import. To test this, we evaluated the siderophore activity of purified dimers in the siderophore-dependent growth condition described previously. We observed that supplementation with either of two dimer metabolites, (DHBS)_2_ or G_2_-(DHBS)_2_, restored bacterial growth in iron-deficient conditions, with slower growth kinetics for G_2_-(DHBS)_2_ dimer than those observed for (DHBS)_2_ dimer and trimers (Figs. 4 and 7*A*). DHBS production was generated from (DHBS)_2_ supplementation and catabolism only (Fig. 7, *B*–*E*). Glucosylated *N*-DHBS (G_1_-DHBS), which was expected to be generated from G_2_-(DHBS)_2_ hydrolysis, was poorly resolved in the LC–MS/MS conditions used here, likely because its high hydrophilicity renders it poorly resolved in reversed-phase liquid chromatography. Together, these data are consistent with siderophore activity by both C-glucosylated and nonglucosylated dimers.

## Discussion

Multiple bacterial siderophore systems release exometabolites in addition to their canonical biosynthetic end products. Here, we find that UPEC have the potential to hydrolyze Ent trimers to recover raw materials for new biosynthesis, yet limit this process to instead generate and secrete incompletely hydrolyzed Ent (dimer), which is released as a siderophore. This suggests a bacterial “choice” between complete hydrolysis to maximize catabolic reclamation of biosynthetic substrates and incomplete hydrolysis to generate a dimeric catabolite that retains siderophore activity. The former lowers the biosynthetic cost of new trimer biosynthesis, whereas the latter yields another siderophore. The balance between these fates (complete or partial hydrolysis) may reflect evolutionary adaptation or, possibly, active regulation.

Siderophore function, as classically understood, is a metabolically costly process in which siderophore biosynthesis and secretion occurs because there is a chance some of these siderophores will diffuse back as iron complexes to support nutritional demands. For Ent and related siderophores, iron release requires hydrolysis by intracellular esterases, suggesting a diminished return on biosynthetic investment compared with siderophores that are nondestructively “recycled” and resecreted (37, 47). The aforementioned results suggest that a more nuanced situation has evolved in which trimer hydrolysis proceeds only to the extent necessary for iron release so that a catabolite may be secreted for additional rounds of siderophore-mediated iron delivery. The growth-promoting siderophore activity of dimers observed here is supported by a previous report of (DHBS)_2_-mediated ^55^Fe localization to *E. coli* (59). Additional supportive evidence was reported for iron-dependent growth of *Campylobacter jejuni*, an Ent nonproducer that uses (DHBS)_2_ from *E. coli* as a siderophore in an example of siderophore “piracy” by this organism (60). The siderophore activity of dimers is thus associated with another example of metabolic cost avoidance.

Although not measured in the present work, it is possible that the loss of a catechol group lowers the iron (III) affinity of Ent-associated dimers relative to trimers, representing a possible trade-off between metabolic efficiency and effector function. A 1:1 dimer–iron complex provides catechol hydroxyl ligands for four of the six iron (III) coordination sites. This tetradentate coordination is observed for other siderophores such as pyochelin from *Pseudomonas aeruginosa* (61, 62) and azotochelin from *Azotobacter vinelandii* (63). Despite this possible drop in affinity relative to Ent (*K*_*d*_ ≈ 10^−52^ M), we observed comparable iron acquisition capability by (DHBS)_2_ dimers. Affinity differences between dimer and trimers could be consequential if associated with differential iron (III) scavenging from complexes encountered in tissue, urine, or other microenvironments. The entire series of *ent*-associated exometabolites, not trimers alone, should be considered in future studies of iron sequestration mechanisms in human and animal hosts.

Incomplete trimer hydrolysis by UPEC suggests that inefficiency in siderophore esterase and peptidase systems has evolved to support dimer-associated iron acquisition. The extent of hydrolysis may vary with the specific trimer and the hydrolases recruited during iron recovery. We found that siderophore-null UTI89 consumed purified Ent without significant monomer or dimer secretion, whereas purified glucosylated Ent trimers (MGE and DGE) resulted in abundant monomer and dimer formation. It remains unclear whether these differences reflect higher order metabolic interactions or unappreciated regulatory process affecting hydrolase activity, possibly responsive to Ent C-glucosylation. Complete hydrolysis of C-glucosylated trimers could be evolutionarily disfavored because of the likely inability to use C-glucosylated DHB as an Ent biosynthetic pathway. Lin *et al.* (47) previously reported that purified Fes and IroD can hydrolyze Ent trimer to produce DHBS monomer and (DHBS)_2_ dimer, and IroE can hydrolyze Ent to produce (DHBS)_2_ dimer.

We used a *tonB* deletion mutant, rather than an outer membrane transport mutant, to assess the effect of siderophore import because of uncertainty over which of the many TonB-dependent transporters import which of the many Ent-associated products investigated here. Among *E. coli*, FepA, IroN, Cir, and Fiu have, to date, been described to import catechols, though their specificity is not completely defined. Among these, only FepA is conserved among all *E. coli.* and is known to mediate ferric Ent import (64, 65), whereas IroN is known to mediate import of glucosylated Ent trimers (66). Cir and Fiu have been demonstrated to mediate Ent breakdown product import (67, 68, 69, 70, 71). Monomeric DHBS-iron(III) complex import has been described by Fiu, FepA, and Cir in *E. coli* (71) and by IroN and FepA receptors in *Salmonella typhimurium* (72). As for Ent dimers, (DHBS)_2_ is found to be taken up by *E. coli* (59), though its specific uptake routes are unclear. Recently, the relevance of nontrimer catechol uptake is exemplified by the clinical antibiotic efficacy of β-lactam agents containing one iron-chelating monomeric catechol moiety, such as cefiderocol (73, 74, 75). The substrate specificity for these transporters and their relationship to the network of Ent-associated exometabolites described here is incompletely understood. Further investigation of this may yield deeper functional insights into Ent system function.

In conclusion, the exometabolite network described here is consistent with a series of regulatory and functional adaptations that minimize costs of Ent-mediated iron delivery in *E. coli* cells. Ent biosynthesis, a metabolically costly process, is activated under iron-restricted conditions by ferric uptake regulator repressor regulation. At low bacterial density, *E. coli* have the ability to render Ent a private good, available only to the producing organism, and minimizing diffusional loss (76). Submaximal siderophore hydrolysis in UPEC to release dimers extends the iron delivery potential of Ent and its derivatives. Together, these results are consistent with a biochemical network connecting intracellular and extracellular *E. coli* metabolomes to cost-effectively support iron-dependent growth. These findings may help explain why Ent expression can be sustained as the universal siderophore system in urinary *E. coli* isolates. Aspects of this network may be useful in devising new antimicrobial therapeutics for UPEC and related bacteria.

## Experimental procedures

### Bacterial strains and culture conditions

We examined exometabolite production, consumption, and use with the well-characterized cystitis-derived model UPEC strain UTI89 and its previously described isogenic mutants UTI89Δ*entB*, UTI89Δ*tonB*, and UTI89Δ*entBΔybtS* (Table S3) (11, 13, 21, 77). UPEC strain CFT073 was used for bacterial secondary metabolite production because of its high yield of C-glucosylated products (Table S3) (11, 13). Bacterial cultures were grown from single colonies in LB broth for overnight under 37 °C, washed with PBS, back-diluted 1:1000 into filter-sterilized M63 minimum media, inoculated with 200 μl into 96-well microplates, and incubated under 37 °C for in the indicated assays. Experimental cultures were conducted in M63 minimum media containing 0.2% glycerol as a carbon source and 10 μg/ml nicotinic acid (low iron), with 100 μM FeCl_3_ (high iron), or with 10 μM bovine serum albumin addition (siderophore dependent) (21, 32). Bacterial growth was quantified by the absorbance at 600 nm using a Spectrophotometer (Beckman Coulter, DU-800) or an incubated microplate reader (Tecan Spark).

### Untargeted LC–MS

Untargeted full scan LC–MS profiling was performed to characterize the extracellular metabolome (exometabolome) in media conditioned by UTI89 and UTI89Δ*entB* under low and high iron conditions. Conditioned medium was collected by centrifugation and filtration through 0.22 μM filters with storage at −80 °C. Samples were thawed on ice for LC–MS analysis with a Shimadzu Prominence UFLC-coupled AB Sciex 4000 QTrap mass spectrometer with a Turbo V electrospray ionization source. LC separation was performed on an Ascentis Express phenyl-hexyl column (100 × 2.1 mm, 2.7 μm; Sigma–Aldrich) with solvent A (HPLC-grade water + 0.1% formic acid; Sigma–Aldrich) and B (90% acetonitrile + 0.1% formic acid; Sigma–Aldrich) at 0.35 ml/min in a 36 min gradient as follows: solvent B increased from 2% to 35% by 23 min, then increased to 98% by 33 min, and finally held steady at 98% for another 3 min. Electrospray ionization -MS was performed in negative ion–enhanced MS mode, scanning from 50 to 1500 *m/z*. A quality control sample was injected first and every 10 samples thereafter to assess instrument stability. MarkerView, version 1.2.0 (Sciex) was used for peak alignment, generating the list of peaks for computational metabolome comparison analysis in the next section (13, 14, 32).

### Computational metabolomic comparison

Exometabolome comparisons between four groups of samples, including UTI89 grown in low and high iron media (wildtype and wildtype + Fe, respectively) and the Ent-null mutant UTI89Δ*entB* in low and high iron media (*entB* and *entB* + Fe, respectively), were performed on a combined computational model consisting of an sPCA followed by a logistic regression classification. The computation was performed in R and Python, using the scikit-learn module and mixOmics package, respectively (78, 79, 80, 81). Of note, sparsity penalization was enforced in the PCA dimensionality reduction step to prevent overfitting for this metabolome metadata consisting of much higher component dimensions than the number of samples (82, 83). The iron-responsive submetabolome in UTI89 extracellular space was identified by the loading analysis of all identified metabolites.

### Product ion scan and targeted LC–MS/MS

Product ion scan measurements were conducted to characterize chemical structures of the 10 Ent-associated molecules. The LC separation as aforementioned but with a flow of 0.5 ml/min and a 16 min gradient as follows. Solvent B increased from 5% to 56% by 10 min, then increased to 98% by 12 min, and finally held steady at 98% for another 4 min. MS/MS product ion spectra of each negative ion was obtained in the enhanced product ion mode (84, 85). Targeted LC–MS/MS MRM analyses were performed to validate the identities of 10 Ent-associated metabolites that were determined by the full-scan comparative metabolomic analysis as described previously. MRM parameter protocols (Table 1) were established based on the results of product ion scan for each of the 10 targeted Ent-associated metabolites (11, 13, 14, 21).

### Exometabolite purifications

Ent-associated exometabolites were generated by growing CFT73 in M63/0.2% glycerol medium supplemented with DHB (Sigma–Aldrich) and 100 μM dipyridyl at 37 °C for 18 h. Culture supernatant was collected and separated by four consecutive steps, including a DEAE-sepharose resin (Sigma), an Amberlite XAD16N resin (20∼60 mesh; Sigma), an Kromasil Eternity 5-PhenylHexyl column (250 × 4.6 mm, 5 μm; Nouryon), and an Ascentis Express Phenyl-Hexyl column (100 × 4.6 mm, 2.7 μm; Sigma–Aldrich) to achieve the purification of five Ent-associated molecules, including Ent, MGE, DGE, [(DHBS)_2_, and G_2_-(DHBS)_2_, as previously described (11, 47). Culture supernatant was first applied to a methanol (20%)-conditioned DEAE-sepharose column (Sigma). The column was washed with water and then eluted with 7.5 M ammonium formate. The DEAE eluate was supplemented with 120 mM sodium dithionite, incubated with methanol-conditioned Amberlite resin (XAD16N; Sigma–Aldrich) overnight, and eluted with 100% methanol. The eluate was concentrated in a rotatory evaporator (R-100 Rotavapor; BUCHI), lyophilized (Labconco), resuspended in HPLC-grade water plus 0.1% formic acid, and further purified on a Bio-Rad BioLogic DuoFlow 10 system equipped with a QuadTec UV–Vis detector and a Kromasil Eternity-5-PhenylHexyl column (Sigma–Aldrich). The Kromasil column was run at 0.30 ml/min with HPLC-grade water plus 0.1% formic acid (solvent A) and acetonitrile plus 0.1% formic acid (solvent B) using gradient as follows. Solvent B held steady at 2% for 1.0 ml, then increased to 15% over 1 ml, then increased to 52% over 40 ml, and finally increased to 100% over 1 ml. The DuoFlow elute was finally separated by another Ascentis Express Phenyl-Hexyl column in a Shimadzu Prominence UFLC system coupled with an SPD-M20A Prominence Diode Array detector. In order to purify the compounds with different properties, the LC separation was performed by injecting solvent A (HPLC-grade water + 0.1% formic acid; Sigma–Aldrich) and B (90% acetonitrile + 0.1% formic acid; Sigma–Aldrich) at 0.5 ml/min with a 44 min gradient under two scenarios as follows. Solvent B increased from 2% to 35% or 44% by 35 min, then increased to 98% B by 38 min, and finally held steady at 98% for another 6 min. Fractions containing purified molecules were measured *via* UV–Vis detection at 319 nm, pooled together, dried down by lyophilization, and stored in −80 °C freezer. On day of use, samples were resuspended in HPLC-grade water plus 0.1% formic acid, and concentrations were calculated by Beer–Lambert law using UV–Vis absorbances at 319 nm with an extinction coefficient of 11,200 M^−1^ cm^−1^. Purity was confirmed by targeted LC–MS/MS measurements (11, 86, 87, 88).

### Exogeneous DHB for synthesizing Ent in isotope-labeling assay

To determine whether UPEC can synthesize Ent from DHB that is not immediately generated by endogenous DHB biosynthesis (40, 41, 89), we grew UTI89 from single colonies in LB broth for 12 h at 37 °C, washed with PBS, back-diluted 1:1000 into ^13^C_3_-glycerol M63 minimum media with or without the supplement of 200 μM ^12^C-DHB in a 96-well plate, and grown at 37 for 24 h. Targeted LC–MS/MS of Ent was conducted to monitor the incorporation of ^12^C from incorporation of unlabeled DHB (Table S2) by comparing ^13^C-substituted Ent isotopologs into which 0, 1, 2, or 3 ^12^C_7_-DHB were incorporated.

### Statistical methods

GraphPad Prism 9.0 (GraphPad Software, Inc) was used to generate graphs and perform statistical analysis in this study. We used the unpaired two-tailed *t* test for comparisons between two groups and one-way ANOVA for multigroup comparisons. *p* < 0.05 was considered statistically significant.

## Data availability

The computer codes for the analyses in this study are available in Github (https://github.com/QL5001/EntMetabolome_script; branch name: main; commit ID, e555df2). All other data generated and analyzed in this study are included in the published article and supporting information.

## Supporting information

This article contains supporting information (11, 13, 14, 21, 46, 47, 51, 86, 90, 91, 92).

## Conflict of interest

The authors declare that they have no conflicts of interest with the contents of this article.
